# Psychometric properties of the culturally adapted 10-item Hopkins Symptom Checklist (HSCL-10-SW) anxiety subscale for southwestern Madagascar – CORRIGENDUM

**DOI:** 10.1017/gmh.2026.10214

**Published:** 2026-05-19

**Authors:** Hervet J. Randriamady, Manasi Sharma, Rocky E. Stroud, Aroniaina M. Falinirina, Romario, Madeleine Rasoanirina, Nadège V. Volasoa, Frédéric Déclerque, Marc Y. Solofoarimanana, Jean C. Mahefa, Hanitra O. Randriatsara, Karestan C. Koenen, Christopher D. Golden

The authors regret that the article was published with two errors.

On page 7, the sentence in the article read: “Therefore, discriminant validity between Depression and Fear Anxiety factors was not conclusive.” The correct sentence should be “Therefore, discriminant validity between Depression and Fear Anxiety factors was conclusive.”

On page 9, the sentence in the article read: “This result was not consistent with Kayaa et al.’s (2018).” This should read: “This result was not consistent with Kayaa et al.’s (2008).”

The article has been corrected.

## References

[r1] Randriamady HJ, Sharma M, Stroud II RE, Falinirina AM, Romario, Rasoanirina M, Volasoa NV, Déclerque F, Solofoarimanana MY, Mahefa JC, Randriatsara HO, Koenen KC and Golden CD (2026) Psychometric properties of the culturally adapted 10-item Hopkins Symptom Checklist (HSCL-10-SW) anxiety subscale for southwestern Madagascar. Cambridge Prisms: Global Mental Health 13, e79. 10.1017/gmh.2026.10185 42064326 PMC13125271

