# Supplementary material for: Psychometric properties of the culturally adapted 10-item Hopkins Symptom Checklist (HSCL-10-SW) anxiety subscale for southwestern Madagascar
Source: Glob Ment Health (Camb). 2026 Mar 30;13:e79. doi: 10.1017/gmh.2026.10185 (PMC13125271; doi:10.1017/gmh.2026.10185)
Supplement: Randriamady et al. supplementary material [file S205442512610185Xsup001.docx]

**SUPPLEMENTAL**

**Table S1: Modification indices with correlated disturbance terms:**

| Disturbance term covariances |  | Estimated parameter change | |
| --- | --- | --- | --- |
|  | MI | unstandardized | standardized |
| ${D_{HSCL-11-SW*}}$ 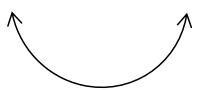 $D_{HSCL-12-SW*}$ | 25.599 | -0.254 | -0.374 |
| ${D_{HSCL-13-SW*}}$ 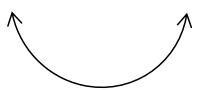 $D_{HSCL-10*}$ | 13.657 | 0.244 | 0.401 |
| ${D_{HSCL-6*}}$ 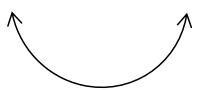 $D_{HSCL-7*}$ | 13.228 | -0.292 | -0.407 |
| ${D_{HSCL-3}}$ 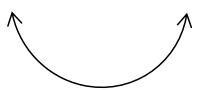 $D_{HSCL-6*}$ | 10.445 | -0.214 | -0.355 |

**Note.** Modification Indices = MI. The curved bidirectional arrows indicate covariances.

**Table S2: Correlations among Depression, Fear Anxiety, Cognitive-Somatic Anxiety factors (Model 2, N=809)**

| **Correlations between constructs** | $\rho$ | **90 % CI** |
| --- | --- | --- |
| Depression 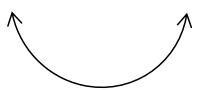 Fear Anxiety | ${\rho_{1}}$= 0.571 | [0.544-0.598] |
| Fear Anxiety 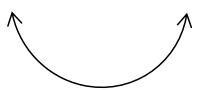 Cognitive-Somatic Anxiety | ${\rho_{2}}$= 0.624 | [0.592-0.656] |
| Depression 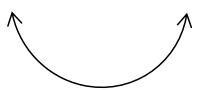 Cognitive-Somatic Anxiety | ${\rho_{3}}$=0.923 | [0.895-0.952] |

**Note.** The one-factor PHQ-8 model was used to measure Depression.
